# A practical comparison of methods for detecting transcription factor binding sites in ChIP-seq experiments

**DOI:** 10.1186/1471-2164-10-618

**Published:** 2009-12-18

**Authors:** Teemu D Laajala, Sunil Raghav, Soile Tuomela, Riitta Lahesmaa, Tero Aittokallio, Laura L Elo

**Affiliations:** 1 Turku Centre for Biotechnology, FI-20521 Turku, Finland; 2 Turku Graduate School of Biomedical Sciences, FI-20520 Turku, Finland; 3 Immune Disease Institute, Harvard Medical School, Boston, USA; 4 Department of Mathematics, University of Turku, FI-20014 Turku, Finland

## Abstract

**Background:**

Chromatin immunoprecipitation coupled with massively parallel sequencing (ChIP-seq) is increasingly being applied to study transcriptional regulation on a genome-wide scale. While numerous algorithms have recently been proposed for analysing the large ChIP-seq datasets, their relative merits and potential limitations remain unclear in practical applications.

**Results:**

The present study compares the state-of-the-art algorithms for detecting transcription factor binding sites in four diverse ChIP-seq datasets under a variety of practical research settings. First, we demonstrate how the biological conclusions may change dramatically when the different algorithms are applied. The reproducibility across biological replicates is then investigated as an internal validation of the detections. Finally, the predicted binding sites with each method are compared to high-scoring binding motifs as well as binding regions confirmed in independent qPCR experiments.

**Conclusions:**

In general, our results indicate that the optimal choice of the computational approach depends heavily on the dataset under analysis. In addition to revealing valuable information to the users of this technology about the characteristics of the binding site detection approaches, the systematic evaluation framework provides also a useful reference to the developers of improved algorithms for ChIP-seq data.

## Background

Chromatin immunoprecipitation (ChIP) enables the identification of *in vivo *protein-DNA interactions under a given condition or in a particular cell type. An important application is the detection of transcription factor binding sites to characterize the regulatory networks controlling, for instance, various cellular processes or physiological states. The high-throughput ChIP techniques are based on identifying on a global scale the sequences and genomic locations of the immunoprecipitated DNA fragments that are bound by the transcription factor of interest. The most common approach until now has been to hybridize the DNA fragments to a tiling microarray (ChIP-chip) [1]. However, the fast development of the next-generation massively parallel sequencing technologies, which enable the direct sequencing of the DNA (ChIP-seq), has recently challenged the microarray-based experiments, providing an alternative particularly useful to study organisms with less well characterized genomes [2,3].

ChIP-seq involves the sequencing of the ChIP-enriched DNA fragments for ~30 bp from their ends. These short sequence reads (tags) are then aligned to a reference genome and binding sites are identified on the basis of their significant accumulation at particular genomic loci using various peak detection algorithms [4]. In addition to a list of genomic regions predicted to be bound by the transcription factor, each potential binding site is typically characterized by the number of reads it contains or the height of the peak determined by the number of overlapping reads. The predicted binding regions can then be used for downstream analyses, such as motif discovery and annotation, to provide further functional context for the biological interpretation.

Together with the development of the ChIP-seq technology, a burst of methods has been introduced for analysing the resulting datasets. In general, these binding site detection algorithms can be divided into window-based approaches, which first define the start and end of a candidate region and then count the number of reads within it (e.g. CisGenome [5]), or overlap-based approaches, which first identify the peaks on the basis of local maxima over read overlaps and then set the start and end of the corresponding candidate region (e.g. FindPeaks [6]). In addition, a method based on hidden Markov models (HMM) is available, which describes the read accumulation along the genome as a sequence of two different states: binding sites and background [7].

The ChIP-seq experiments, in turn, can be divided into those involving negative control samples and those that contain only ChIP samples. The main difference in analysing these datasets is that the former allow the statistical significance of the peaks to be estimated on the basis of an empirical background. Accordingly, some of the current algorithms are applicable only when a control sample is available (e.g. QuEST [8]), while some are not designed to incorporate control data (e.g. GeneTrack [9]); however, most of the algorithms provide different variants depending on the type of the data (e.g. MACS [10]).

While several recent publications have introduced new algorithms for ChIP-seq data analysis and the authors have shown improved performance over the previous approaches in selected datasets, an independent comparison of their relative performance using common datasets is still lacking. At the moment, there is only limited information on the use of the different approaches in practical applications; what are their relative merits and limitations; or are there actually large differences between the methods.

The present study compares systematically the performance of the different peak detection algorithms in predicting transcription factor binding sites in ChIP-seq data. In particular, we compare nine publicly available algorithms that represent the current state-of-the-art of the field: PeakFinder [2], GeneTrack [9], FindPeaks [6], SISSRs [11], QuEST [8], MACS [10], CisGenome [5], PeakSeq [12], and Hpeak [7] (Table 1). The focus here is on evaluation measures that are of practical interest to researchers when analysing their datasets. First, we point out potential differences in the biological conclusions made when different methods are applied. Then, we evaluate the detections in terms of their reproducibility across biological replicates. Finally, we investigate the overlap of the predicted binding sites with the corresponding sequence motifs or binding regions confirmed in independent qPCR experiments. Since our aim is to give an objective and practical assessment of the algorithms, we apply each method using their default parameters and following the instructions provided in the software manuals. Systematic comparisons are shown in four diverse datasets (Table 2). In addition to three publicly available datasets, against which any future algorithm can be easily compared, we also include into the evaluations our in-house ChIP-seq data on the binding of the STAT6 transcription factor to account for the fact that some of the datasets have been used to train particular algorithms and their default parameters may therefore be ideal for these data.

**Table 1:** Peak detection algorithms investigated in the present study

Algorithm	Availability	Reference	Type	Background model
PeakFinder 2.0.1	http://woldlab.caltech.edu/html/chipseq_peak_finder	[2]	S, C	none

GeneTrack 1.0.1	http://code.google.com/p/genetrack/	[9]	S	none

FindPeaks 3.1.9.2	http://www.bcgsc.ca/platform/bioinfo/software/findpeaks/	[6]	S	uniform

SISSRs v1.4	http://sissrs.rajajothi.com/	[11]	S, C	Poisson/ control sample

QuEST 1.0	http://mendel.stanford.edu/sidowlab/downloads/quest/	[8]	C	control sample

MACS 1.3	http://liulab.dfci.harvard.edu/MACS/	[10]	S, C	local Poisson/ control sample

CisGenome v1	http://www.biostat.jhsph.edu/~hji/cisgenome/	[5]	S, C	negative binomial/ control sample (binomial)

PeakSeq v1.01	http://www.gersteinlab.org/proj/PeakSeq/	[12]	C	local Poisson and control sample (binomial)

Hpeak 1.1	http://www.sph.umich.edu/csg/qin/HPeak/	-	S, C	hidden Markov model

The column Type indicates whether the method is applicable to a single sample analysis (S) or a two-sample analysis involving a control sample (C).

**Table 2:** ChIP-seq samples analysed in the present study

Sample	Cell type	Binding motif (Genomatix)	Reads (million)	Reference
NRSF	Jurkat	V$NRSF.01	2.3	[2]

Control	Jurkat	-	1.7	[2]

NRSF mono	Jurkat	V$NRSF.01	5.4	[8]

NRSF poly	Jurkat	V$NRSF.01	8.8	[8]

Control	Jurkat	-	17.4	[8]

FoxA1	MCF7	V$HNF3.01	3.9	[10]

Control	MCF7	-	5.9	[10]

STAT6	Th2 1 h	V$STAT6.01	3.0	Elo *et al*. (unpublished)

STAT6	Th2 4 h	V$STAT6.01	2.7	Elo *et al*. (unpublished)

STAT6	Thp	V$STAT6.01	3.2	Elo *et al*. (unpublished)

## Methods

### Peak detection algorithms

A short summary of the algorithms being compared in the present study is given below. For more details, the reader is referred to the original publications or the software websites (Table 1). When applying the algorithms, we followed the instructions and recommendations given in the software manuals as closely as possible to provide a fair evaluation. In particular, the default values for the parameters were used, which is likely to be the choice of most average users.

*PeakFinder *identifies candidate binding sites as aggregations of *k *or more ChIP reads not separated by more than *n *bp (default 75) and with an additional requirement that at least 5 of the reads are overlapping. If a control sample is available, then it is further required that there is at least an *m*-fold enrichment (default 5-fold) of reads in the ChIP sample over the control within the same boundaries, and the data are recommended to be normalized into numbers on a per-million reads basis. Following the default settings, we used *k *= 20 when a ChIP sample was analysed without a control, and *k *= 8 when a control sample was utilized. PeakFinder does not provide any estimates for false discovery rate (FDR).

*GeneTrack *applies a Gaussian smoothing procedure to represent the read densities along the chromosomes. More specifically, a normal distribution with a specified standard deviation (default 20) is utilized. Peaks are identified by finding the local maxima in the smoothed data. The results are reported for both strands separately as well as for their composition (sum of the individual strands). An optional exclusion zone parameter allows to determine a distance, within which only a single peak is identified. Since no guidance is provided on how to optimally utilize the strand information nor how to define the exclusion zone parameter in the context of transcription factor binding, we focused here on the composite-strand detections with the exclusion zone set to 0. To reduce the large number of identifications made due to these choices (even millions of small peaks), we only considered peaks having a score over 7, which is a compromise between the default overlap and count requirements of PeakFinder. GeneTrack does not provide any options to estimate the FDR levels of the detections.

*FindPeaks *extends each aligned read directionally to an estimated length of the DNA fragment. The candidate binding sites are then identified on the basis of overlapping fragments (peak height) along the genome. The FDR is estimated using a Monte Carlo simulation, in which random locations are repeatedly generated for the reads and the number of peaks at a given height threshold in the randomized data is divided by the number of peaks observed at the same threshold in the real experimental data. As suggested in the FindPeaks manual, the reads were filtered to remove duplicate hits, the fragment length distributions were estimated by assuming a triangle-based distribution with a median value of 200, while enabling directional peak detection, peak trimming and subpeak detection modules. Peaks at FDR < 0.05 were identified.

*SISSRs *(Site Identification from Short Sequence Reads) also starts by extending the reads to the estimated length of the DNA fragment, similarly as FindPeaks. It then scans the genome using a window of width *w *(default 20) and, for each window, it subtracts the number of antisense reads from the number of sense reads (called net tag count). Binding sites are identified as transition points of this count from positive to negative, provided that the number of directional reads on each side of the inferred binding site is at least *n *(default 2). A Poisson background or a negative control sample, if available, is used to estimate the FDR, which is determined as the ratio of the number of peaks indicated by the background model to the number of peaks observed in the real data. The default FDR threshold of 0.001 was applied.

*QuEST *(Quantitative Enrichment of Sequence Tags) shares with GeneTrack the idea of applying a Gaussian kernel separately to both strands (default bandwidth 30). The major difference is that QuEST estimates a peak shift between the strands before aggregating the results. Moreover, while GeneTrack does not provide any practical guidance on how to choose a suitable threshold for a peak, QuEST determines peak calls based on the properties of separate control data. In particular, the ratio of the detected peak to the background is required to exceed a specified threshold value, called the rescue ratio (default 10). If the negative control has enough reads (at least approximately twice as many as the ChIP sample), then also FDR estimation is provided, in which the control sample is divided into two parts to mimic a random two-sample comparison and FDR is estimated as the ratio between the number of peaks detected in this random comparison and the number of peaks detected in the actual comparison in the real data with the same parameters. QuEST does not support analyses without controls.

*MACS *(Model-based Analysis of ChIP-Seq) is another algorithm besides QuEST that takes advantage of the observed bimodal enrichment patterns of binding sites by empirically modelling the shift size. Unlike QuEST, however, it is applicable also without negative controls. To capture local biases in the genome, a dynamic Poisson distribution is used to model a local background. Candidate peaks with *p*-values below a user-defined threshold (default *p *< 10^-5^) are identified. If negative control data are available, then also FDR is estimated. More specifically, the same parameters are used to determine the number of ChIP peaks over the control sample and the number of control peaks over the ChIP sample, and the FDR is defined by dividing the latter by the former. In the present study, the default *p*-value threshold was applied.

*CisGenome *scans the genome with a sliding window (default width 100) and identifies regions with enriched read counts. The FDRs are estimated assuming that the background read occurrence follows a negative binomial distribution, which was suggested to provide a better fit to the real data than the (global) Poisson distribution [5]. The FDR is determined by calculating the ratio between the number of peaks expected by the null model at a particular cutoff level and the observed number of peaks detected at the same level. In the present study, we identified regions at FDR < 0.05, similarly as with FindPeaks. In the presence of negative control data, a binomial model is used to determine whether the read enrichment relative to the control is significant. The distribution of the window-based read counts in the ChIP sample is compared with what is expected by a binomial distribution given the total count in the ChIP and control samples. The default FDR threshold of 0.1 was considered. The directionality of the reads was utilized to refine the peak boundaries.

*PeakSeq *first constructs a similar read density map as FindPeaks by extending the reads directionally to the average length of the DNA fragments and determining their overlap along the genome. Similarly as in MACS, a candidate set of peak regions is then identified in a ChIP-sample assuming a local Poisson background, taking into account also the variability in genomic mappability. Finally, the significance of enrichment of these candidates is determined relative to a negative control sample using a binomial distribution, similarly as in CisGenome, assuming that under the null hypothesis the reads should occur with equal likelihood from the ChIP and control sample. To control FDR, the obtained *p*-values are adjusted using the procedure of Benjamini and Hochberg [13]. The default FDR threshold of 0.05 was used.

*Hpeak *identifies peaks using a two-state hidden Markov model (HMM), whose states correspond to the binding sites and the background. The emission probabilities are described by two different Poisson distributions. The significance of enrichment of the peaks is adjusted using the Bonferroni correction for multiple testing. The default significance threshold of 0.001 was applied.

### Datasets

To compare the peak detection methods across diverse studies, four different datasets were considered, which measure the binding of three human transcription factors, STAT6, NRSF (two datasets) and FoxA1 (Table 2). The STAT6 data are from our unpublished study, whereas the three other datasets are from publicly available sources. All the datasets were sequenced using the Illumina's Solexa sequencing technology [14].

Our in-house data measures the binding of STAT6 (signal transducer and activator of transcription 6) transcription factor in human T helper (Th) cells. One of the samples is from naïve Th precursor (Thp) cells, whereas the other two samples are activated and induced by IL4 to polarize towards the Th2 subtype (measurements at 1 h and 4 h after polarization). Further details about the data and the biological conclusions will be presented elsewhere (Elo *et al*., unpublished).

Two of the public datasets measure the binding of the transcription factor NRSF (neuron-restrictive silencer factor) in Jurkat T cells. The first NRSF dataset is from the study of [2] and was downloaded from the Illumina website [15]. It contains one NRSF ChIP sample and a corresponding negative control. The other NRSF dataset is from the study of [8] and was downloaded from the QuEST website. It contains a monoclonal and a polyclonal NRSF ChIP sample as well as a control input sample. The FoxA1 data measures the binding of FoxA1 (hepatocyte nuclear factor 3α) transcription factor in MCF7 cells. It contains one FoxA1 ChIP sample and one input control sample. The data were downloaded from the MACS website [10].

The STAT6 read data were aligned to the human reference genome (NCBI v36) using the short oligonucleotide alignment program SOAP [16]. The publicly available datasets were already aligned in the original studies and these preprocessed data were used. Only uniquely mapped coordinates were considered for further analysis. The number of aligned reads for the different samples varied from 1.7 to 17.4 million (Table 2).

### Evaluation procedure

The binding site detections with each algorithm were made using the individual ChIP samples alone (all except QuEST and PeakSeq which were not applicable without a control) as well as with the corresponding negative controls (all except GeneTrack and FindPeaks which do not provide such an option). Thus, in total, fourteen peak lists were generated for each ChIP sample. In the following, we refer to the results involving the control data by adding an additional C to the name of the method (e.g. the two alternative modes of PeakFinder are referred to as PeakFinder and PeakFinderC). In the STAT6 data, the Thp sample was used in place of a negative control, although it may contain also some true biological signal. This choice was motivated by the assumption that the relevant binding events do not yet occur in the Thp samples [17].

First, we evaluated the reproducibility of the methods across biological replicates in the NRSF datasets. This gives indications of the robustness of the methods, as the biologically relevant binding sites are expected to be detected in each replicate. The reproducibility between two peak lists was defined in terms of their overlap, following the approach of [18] to deal with the ambiguity that a peak in one dataset may overlap multiple peaks in another dataset. More specifically, the peak regions from the two peak lists under comparison were first merged into a union set of *n *regions. These were then compared to the original two lists to determine the number of regions identified in both lists *n*_1_. Finally, the reproducibility was defined as *n*_1_/*n*, which obtains the value 1 if all the peaks are overlapping and the value 0 if none of the peaks overlaps.

Second, the detected peaks were evaluated in terms of their overlap with high-scoring sequence motifs. It is known that transcription factors exhibit binding sequence specificities, and hence, the genomic coordinate of a peak should be marked by a canonical binding site motif [8]. Since such motifs are known for the three transcription factors considered in this study (Table 2), they were used as an external source of information to assess the performance of the peak detection algorithms. For each sample, the overlap of the predicted binding sites with the known motifs was determined using the Genomatix RegionMiner tool with default settings [19]. To deal with the potential biases caused by the different peak widths, the motif *z*-scores were considered, which measure the overrepresentation of a motif against an equally sized sample of the genomic background. With GeneTrack and QuEST, which reported only a single coordinate for each peak, 200 bp sequences from around the peak calls were utilized, similarly as in [8]. These same regions were also used in the other overlap calculations.

Finally, the performance of the peak detection algorithms was evaluated with respect to regions that were confirmed in independent qPCR experiments to be bound by the particular transcription factor (true positives) as well as regions that did not show binding (true negatives) in these experiments. For NRSF, we used the 83 true positives and 30 true negatives given in [20]. For FoxA1, 26 true positives and 12 true negatives were obtained from [21]. For STAT6, we have tested a total of 25 regions at various levels of read enrichment selected on the basis of manual inspection to verify the ChIP-seq results at 4 h (Additional file 1). Of these regions, 17 were confirmed to be bound by STAT6. The known true positive and true negative regions in each data allowed us to assess the performance of the methods in terms of their receiver operating characteristics (ROC), which consider both the sensitivity and specificity of the detections. Additionally, an empirical FDR estimate was calculated as the proportion of false positives among the identified candidate peaks.

## Results

### Characteristics of the detected binding regions

**Figure 1:**
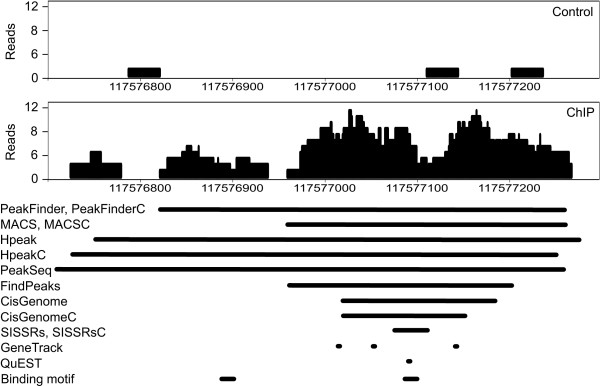
**An example region identified as a STAT6 binding site at 1 h after polarization with IL4**. The same region was identified as a STAT6 binding site with all the fourteen peak detection approaches applied in the present study. The number of overlapping reads (*y*-axis) is shown at each genomic position (*x*-axis). The horizontal bars below the profile illustrate the detected binding regions, as well as the high-scoring STAT6 binding motifs as determined using the Genomatix MatInspector tool.

We first investigated the characteristics of the detected binding sites in terms of an example region in the STAT6 data (Figure 1). Although the same region was detected with all the algorithms, there were marked differences in the region boundaries reported by the different software. In this example, PeakFinder, PeakSeq and Hpeak identified a relatively long region, whereas FindPeaks, SISSRs, MACS and CisGenome reported shorter regions. GeneTrack and QuEST identified only single coordinates without boundary estimates. As GeneTrack was allowed to detect multiple peaks close to each other, three separate detection calls were produced. Notably, the ~100 bp region shared by all of the methods contained also a high-scoring STAT6 sequence motif, suggesting that the detection is likely to be a true binding site. This was further supported by the control Thp sample, which did not show any enrichment of reads in the region, and the detection was finally confirmed with qPCR. Interestingly, another high-scoring binding motif was detected in the lower-enrichment region that was covered only by the PeakFinder, PeakSeq and Hpeak detections but missed with the other algorithms. The correctness of this detection remains to be shown in future experiments. In addition to being an example of a binding site, Figure 1 provides also a representative illustration of the typical bimodality of the peak patterns caused by the fact that short reads are sequenced from both ends of the ChIP DNA fragments.

To get a more general picture of the binding sites identified by the different algorithms, we calculated the average peak width for each NRSF, FoxA1 and STAT6 sample listed in Table 2. In general, PeakFinder and PeakSeq gave the widest regions (median over the samples > 400 bp), whereas SISSRs and CisGenome identified the narrowest peaks (< 100 bp) among the methods that estimate the boundaries. The average differences between FindPeaks, MACS and Hpeak were minor (average peak width ~300 bp), but MACS showed largest variability between the samples. In particular, MACS resulted in narrow peaks (< 100 bp) in our STAT6 data, whereas it produced wider peaks (~300 bp) in the other datasets. The use of a control sample did not dramatically affect the average peak widths with any of the methods.

### Number of identifications

**Figure 2:**
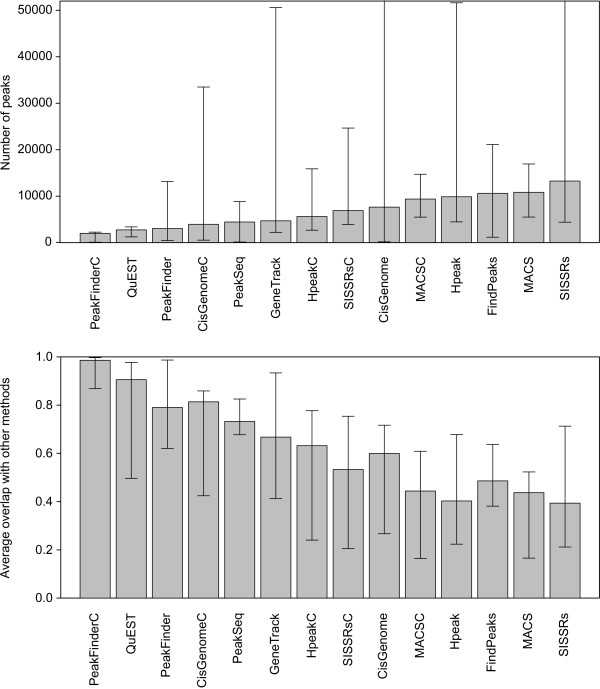
**Numbers and overlaps of the detected peaks**. The upper panel shows the median number of detected peaks across the different ChIP samples and the corresponding minimum and maximum values (error bars). For the clarity of illustration, the maximum values with SISSRs (78634) and CisGenome (78551) are cut out from the figure. The lower panel illustrates the overlap of the detections with a particular method as compared to all the other methods. The median percentage of overlapping peaks is shown together with the minimum and maximum values (error bars).

The number of detected binding sites varied greatly depending on the algorithm (Figure 2, upper panel). PeakFinder and QuEST produced systematically the lowest numbers of detections, whereas FindPeaks, SISSRs and MACS tended to identify a larger number of peaks. GeneTrack, SISSRs, CisGenome and Hpeak showed enormous variability in the numbers of detections even between the samples measuring the same transcription factor. For instance, in the NRSF samples, the number of identifications ranged from ~3000-5000 to over ~30000. The investigation of the average overlap between the algorithms showed that most of the binding sites identified with the methods producing the shortest lists were also detected with the other approaches (Figure 2, lower panel). For instance, nearly all the binding sites identified with PeakFinderC were also identified with most of the other algorithms.

### Differences in biological conclusions

A closer investigation of the binding site detections in the STAT6 data revealed the more practical differences between the algorithms in real applications. Strikingly, approximately half of the algorithms (PeakFinder, PeakFinderC, FindPeaks, CisGenome and PeakSeq) suggested that there were more binding sites at 1 h than at 4 h, whereas another half (SISSRs, SISSRsC, MACS, MACSC, CisGenomeC, Hpeak and HpeakC) identified more binding sites at 4 h (data not shown). The extreme cases were CisGenome/PeakSeq and SISSRs/HpeakC, whose results varied from an ~8-fold decrease to a ~2-fold increase in the numbers of detections from 1 h to 4 h. This example demonstrates how the differences in the peak identification algorithms may lead to a notably variable overall picture of the dataset under analysis, emphasizing the importance of careful assessment of the resulting peak lists. A plausible technical explanation for the differences in the numbers of peak detections in different samples with different read distributions lies in the preferences of the algorithms. For instance, CisGenome and PeakSeq rely strongly on the peak heights, whereas SISSRs and Hpeak consider more the local statistical enrichment patterns. The biological significance of the identified STAT6 binding sites and further analysis of the binding kinetics remains to be addressed in further studies.

**Figure 3:**
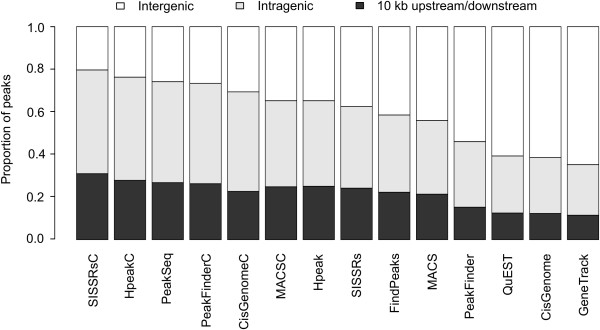
**A representative example demonstrating how biological conclusions may change when different algorithms are applied**. The physical distribution of the binding sites in the STAT6 data is shown at 1 h after polarization with IL4. The binding sites were divided into three categories: 10 kb upstream/downstream of a transcription start/end site, within a gene (intragenic), or over 10 kb from a gene (intergenic). The proportion of binding sites in each category is indicated by the colours.

In addition to the number of detected peaks, another practical question is their physical location in the genome. Accordingly, we divided the detections into groups on the basis of their location within 10 kb upstream/downstream of a transcription start/end site, within a gene (intragenic), or over 10 kb from a gene (intergenic) using the CisGenome software [5]. Again, drastic differences were observed between the algorithms (Figure 3). For instance, GeneTrack, QuEST and CisGenome suggested that only less than 40% of the STAT6 binding sites at 1 h reside within 10 kb of a gene or are intragenic, whereas with PeakFinderC, SISSRsC, PeakSeq and HpeakC the corresponding estimate was over 70%.

### Reproducibility of the detections

**Figure 4:**
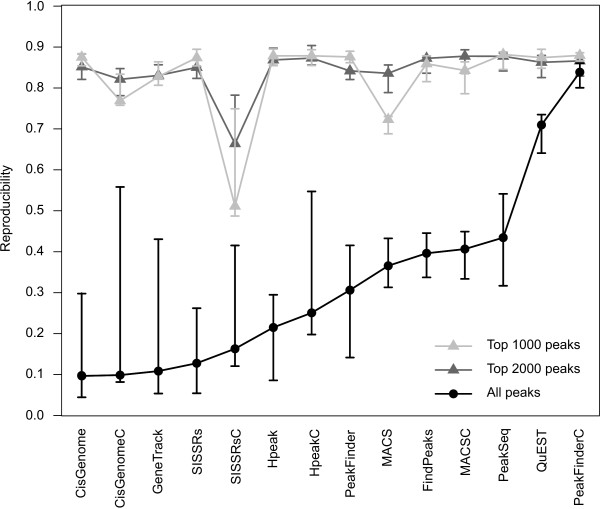
**Reproducibility of the detections across the three NRSF samples**. With each method, the reproducibility was determined by first creating a union set of the detected regions and then assessing which of these regions were specific to only one of the samples under comparison and which were detected in both samples. The median reproducibility is shown together with the minimum and maximum values (error bars).

The two NRSF datasets with biological replicate samples provided an opportunity to assess the performance of the algorithms internally by investigating their ability to reproduce the detections robustly across the replicates. When all the peaks were considered, the best reproducibility was observed with PeakFinderC and QuEST (Figure 4). With each method, the use of a negative control sample improved consistently the reproducibility of the detections. In general, GeneTrack, SISSRs, CisGenome and Hpeak gave the lowest reproducibility values, which could to a large extent be attributed to the fact that huge differences were observed in the numbers of detections across the replicates. With these algorithms, the NRSF polyclonal sample produced markedly more detections than the other NRSF samples (even over a ten-fold number). PeakFinderC and QuEST, on the other hand, identified only a relatively small number of peaks (2000-3000) and the number of peaks was consistent across all the three samples.

When considering only the top 1000 or top 2000 peaks with each algorithm, all the algorithms showed high reproducibility and the overall differences between the methods were typically negligible. Only SISSRsC showed somewhat lower reproducibility values than the other algorithms. The lower reproducibility of MACS with top 1000 peaks disappeared when the top 2000 detections were considered.

### External validation using binding motifs

**Figure 5:**
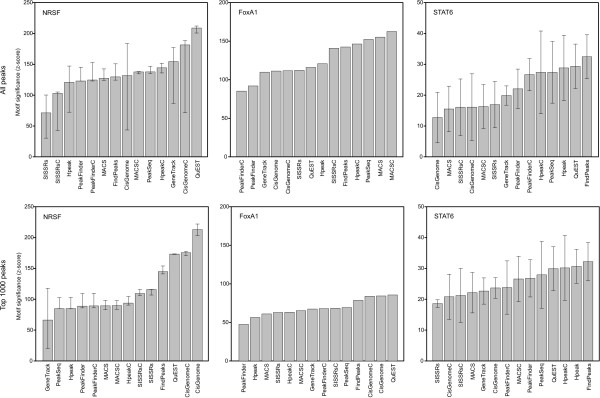
**External validation of the predicted binding sites using binding motifs**. The significance of the overlap of the identified peaks with the corresponding high-scoring sequence motifs (see Table 2 for the motif identifiers) was assessed by the Genomatix RegionMiner software separately for each transcription factor (columns), either when using all the detected peaks (upper panel) or when focusing on the top 1000 peaks only (lower panel). The medians over the ChIP samples (bars) are shown together with the minimum and maximum values (error bars).

A comparison of the detected peaks with high-scoring sequence motifs confirmed that all the algorithms identified binding sites at a highly significant overlap with the corresponding sequence motif (Figure 5). When the whole set of detected peaks was investigated with each algorithm (Figure 5, upper panel), in the NRSF data, the most significant overlap was observed with QuEST, while there were no systematic differences between the other methods. With GeneTrack, SISSRs, CisGenome and Hpeak, however, the variability between the samples was large, the NRSF polyclonal sample performing considerably worse than the other two samples. In the FoxA1 data, MACSC was the best-performing approach in terms of the sequence motifs, whereas PeakFinderC showed the least significant motif overlap. In the STAT6 data, the most significant overlap was observed with FindPeaks, while MACS, which was among the best methods in the FoxA1 data, was among the poorest approaches together with SISSRs and CisGenome.

When focusing only on the top 1000 candidates (Figure 5, lower panel), the best-performing approaches in the NRSF and FoxA1 datasets were QuEST and CisGenome, which produced typically relatively narrow peak regions. This is in line with the observation that all the methods are likely to perform reasonably well when detecting the most prominent peaks (Figure 4), in which case a more specific detection of the actual binding site can be considered as a benefit and is captured also by the motif *z*-score. In principle, a narrower peak width can indicate a better resolution and can be beneficial, for instance, for the discovery of *de novo *binding site motifs [5]. The top list size 2000 produced similar results (data not shown). In the STAT6 data, the overall number of peaks was much lower than in the NRSF and FoxA1 datasets (often even less than 1000 peaks with the default settings), and several algorithms could detect only less than 2000 peaks even if the detection thresholds were lowered from their default values. In the STAT6 data, FindPeaks remained the best-performing algorithm also when the top 1000 detections were evaluated.

### External validation using qPCR

**Figure 6:**
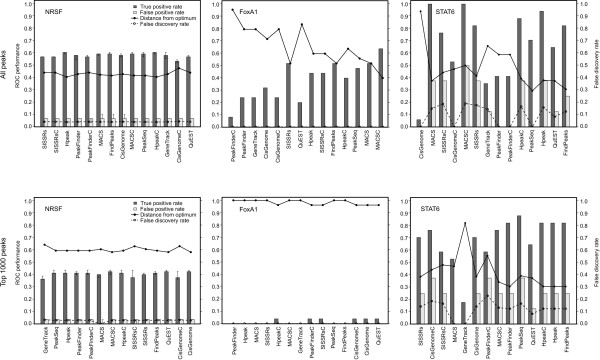
**External validation of the predicted binding sites using qPCR**. The performance of the binding site detection methods as assessed by independent qPCR validations, either when using all the detected peaks (upper panel) or when focusing on the top 1000 peaks only (lower panel). In total, there were 83, 26 and 17 true positives and 30, 12 and 8 negative validation results for NRSF, FoxA1 and STAT6, respectively. The true positive rate (TP, true positives divided by all positives) and the false positive rate (FP, false positives divided by all negatives) are shown by the bars (median, minimum and maximum in the NRSF data), whereas the solid lines illustrate their distance from the optimal performance [(1-TP)^2 ^+ FP^2^]^1/2^. The dashed lines show the empirical estimate of false discovery rate (FDR), calculated as the proportion of false positives among all the identified positives.

To complement the motif overlap evaluations, qPCR-validated regions were used to assess the sensitivity and specificity of the methods (Figure 6). In the NRSF data, all the algorithms performed rather similarly in identifying the validated positive and negative regions both when considering the whole set of detections as well as when focusing on the top peaks only. In the FoxA1 data, the qPCR validations supported further the good performance of MACSC when all the detections were used in the evaluation, while only one of the top 1000 binding sites was included in the set of validated regions. Also in the STAT6 data, the qPCR validations were well in line with the motif analysis results, suggesting the good performance of FindPeaks in these data. Importantly, besides demonstrating the impact of the dataset on the relative performance of the algorithms, the qPCR analyses supported the utility of the motif significance assessments in choosing a suitable algorithm for peak detection in cases in which the binding motif is known. In each dataset, the method with the most significant motif overlap was among the best algorithms in terms of the qPCR validations.

The qPCR validations enabled also to determine an empirical FDR for the detections. In the NRSF data, the empirical FDR was below 0.05 with all the algorithms. In the FoxA1 data, no negatives were detected with any of the algorithms, giving an empirical FDR estimate of 0. In the STAT6 data, the estimated FDR remained typically below 0.2. Notably, however, these values were often markedly higher than those suggested by the background models of the algorithms, supporting the earlier observation that especially the Poisson-based randomization model can severely underestimate the FDRs [22].

## Discussion

The present study evaluated from a practical point of view the performance of the currently available open source software for detecting transcription factor binding sites in ChIP-seq data. A main observation was that the choice of the algorithm may considerably affect the overall conclusions made from the data (Figure 3). Moreover, there was no clear winner among the methods that would have outperformed the other approaches systematically in each dataset. Instead, the choice of the best method was strongly dependent on the data under analysis (Figure 5). While QuEST performed well in the NRSF data, MACS may be a better choice in the FoxA1 data, whereas FindPeaks showed good performance in the STAT6 data. Below we discuss some practical guidelines for the researchers, including (i) the choice of an appropriate algorithm for different study objectives, (ii) the use of a negative control sample, and (iii) the use of empirical validations.

In most of the currently published ChIP-seq studies, the choice of the peak detection algorithm lacks detailed motivation or description (see Table 3 for a set of representative cases). Our comparison demonstrates, however, that this choice warrants careful attention. While most computational methods perform well under some circumstances, their behaviour can vary markedly depending on the dataset under analysis. This is especially true when the aim is to detect all the potential binding sites of a particular transcription factor of interest. If only a small set of best candidate targets are to be detected, then all the methods performed relatively well in our comparisons (Figure 4). To identify the best candidates, the candidate binding positions can be prioritized using the peak magnitude scores or their *p*-values, provided by the peak detection software.

**Table 3:** Recent ChIP-seq studies

Transcription Factor(s)	Organism	Algorithm(s)	Negative control sample	qPCR validations	Reference	Time of publication
Scl/Tal1	Mus musculus	FindPeaks	no	>10	[32]	5/2009

ERalpha	Homo sapiens	FindPeaks (MACS)	yes	>10	[33]	5/2009

STAT1	Homo sapiens	PeakSeq	yes	>100	[12]	1/2009

CTCF	Homo sapiens	SISSRs	no	not reported	[34]	1/2009

Cse4, Ste12	Saccharomyces cerevisiae	PeakSeq	yes	<10	[35]	1/2009

PPARg, RXR	Mus musculus	FindPeaks	no	>10	[36]	11/2008

Stat5a, Stat5b	Mus musculus	MACS	yes	<10	[25]	11/2008

NRSF, SRF, GABP	Homo sapiens	QuEST	yes	not reported	[8]	9/2008

FoxA1	Homo sapiens	MACS (PeakFinder, QuEST, FindPeaks)	yes	not reported	[10]	9/2008

NRSF	Homo sapiens	PeakFinder	yes	>100	[2]	6/2007

Representative examples of recent ChIP-seq studies with publicly available datasets were collected from the Gene Expression Omnibus (GEO). Additionally, the public datasets used in the present study were included. The column Algorithm(s) indicates the algorithm applied in the original study. If other algorithms were also considered, they are listed in the brackets. The column qPCR validations indicates the level of experimental validation in terms of the numbers of validated regions.

When the goal is to identify a comprehensive set of regulatory interactions, the major challenge is to determine a suitable threshold to discriminate true binding sites from background noise. This was exemplified by the large differences in the numbers of peak calls observed with the different approaches (Figure 2). PeakFinder and GeneTrack do not provide any statistical estimates of the FDR, making it difficult to choose an appropriate cutoff. Although the other algorithms estimate also the statistical significance of the detections, the accuracy of the estimation can depend heavily on the choice of the selected null model [5,23]. While the simplest model assumes that the background read density is uniform along the genome and independent between the strands, several authors have observed that the sequenced control samples show highly non-uniform behaviour and, in some cases, their read density patterns are close to those expected from true binding sites [5,22,23]. This can be due to various reasons, such as sequencing and mapping biases, non-specific immunoprecipitation or differences in the chromatin structure [10,24]. Therefore, the use of separate control samples has been suggested [5,8,10,22], and was also supported in our comparisons (Figure 4). If experimentally determined true positive and true negative binding sites are available, then it is possible to calculate also an empirical FDR for the detections (Figure 6).

Besides the inclusion of a control sample, another important decision concerns the type of an appropriate control. At least three types of controls have been considered: a non-immunoprecipitated fragmented DNA sample (input DNA) [12], a ChIP-seq sample using an unspecific antibody (e.g. IgG) [25], or a ChIP-seq sample under a different cellular condition (e.g. without stimulation) [26]. Further study is needed to determine which control sample type provides the best outcome in different algorithms. In addition, as the quality of the immunoprecipitating antibody critically affects the results, the actual ChIP experiment may also be repeated with different antibodies [8,24].

If the binding motif of the transcription factor of interest is known, then it can provide useful information about the relative performance of the different approaches (Figure 5). However, there are also transcription factors that do not require a specific binding motif [27]. Further information about the adequacy of the peak detection methods can be obtained by experimental validations (Figure 6). Since confirmation studies on candidate binding sites are expensive and time-consuming, however, thorough experimental validations are relatively rarely done when reporting large-scale findings. Moreover, it is worth noting that building an appropriate set of true negatives is a difficult task, and it has been suggested that the sets of the previously utilized true negatives may actually contain also true positives despite their low enrichment ratios in the qPCR validations [22]. How to choose the best method directly from the data remains a challenging future research question.

From the point of view of an ordinary user, a major complication of the peak detection software is the typically large number of adjustable parameters. While the default parameters are a natural choice and were applied also by us, they may not be optimal for the particular data under analysis. On the other hand, if an algorithm lacks the possibility to easily adjust the parameters properly, it can be regarded as a weakness of the method. Other critical issues in ChIP-seq data analysis are the memory requirements for the computer and the diversity of the current data formats. The required input formats of the peak detection software as well as their output peak lists are far from being standardized, neither are the output formats produced by the different read alignment software. Further technical challenges include, for instance, the quality of the aligned reads and the required depth of sequencing [22]. Also the interpretation of the results poses its own challenges. Even if a comprehensive and unbiased set of binding sites could be determined with ChIP-seq, the identified sites may not all be functional regulatory elements that have an impact on transcription. Instead, it is possible that several non-functional detections are made as a consequence of biological noise [28,29].

Despite the challenges, the next-generation DNA sequencing has a great potential to accelerate biological and biomedical research by enabling a comprehensive analysis of genomes, transcriptomes and interactomes to be performed routinely without having the resources of large genomic centres [3]. While several issues remain to be solved regarding, for instance, the optimization of the peak detection algorithms, already the current results support the utility of the ChIP-seq technique. In our comparisons, for example, all the algorithms identified binding sites with highly significant overlap with the corresponding known sequence motif (Figure 5), and the most prominent peaks were typically detected robustly across independent experiments (Figure 4). In addition to transcription factor binding, a wide range of other biological phenomena can be investigated, such as chromosome conformation, genetic variation, and RNA expression (RNA-seq) to detect, for instance, differential splicing, microRNA and other non-coding RNAs [3,30,31].

With the growing importance of the technology, rigorous computational approaches to transform the large datasets into biological knowledge are required to truly leverage the potential of these data. Rather than introducing a number of closely related algorithms, it is critical to objectively evaluate their performance to provide practical guidance to the researchers analysing their data and to the developers of the algorithms to evaluate their new ideas. An important future direction is also to effectively integrate ChIP-seq data with other types of datasets, such as those generated in siRNA interference experiments, to improve the detection of target genes.

## Conclusions

ChIP-seq combines chromatin immunoprecipitation with next-generation sequencing to identify *in vivo *protein-DNA interactions on a genome-wide scale. With the increasing popularity of the ChIP-seq technology, a number of algorithms have been presented to analyse the resulting datasets. However, only little is known about their relative merits and limitations in practical applications. Rather than introducing a range of closely related procedures, it is therefore crucial to objectively assess their performance in practice. In the present study, we compare systematically the current state-of-the-art of detecting transcription factor binding sites from ChIP-seq data under a variety of practical research settings, including reproducibility across biological replicates as well as comparisons to high-scoring binding motifs or independent experimental validations. Our results suggest that the optimal choice of the algorithm depends heavily on the dataset under analysis. In addition to providing practical guidance to the users of the ChIP-seq technology, our systematic evaluation framework is also a useful reference to the developers of improved algorithms.

## Authors' contributions

TDL performed the computational testing. SR carried out the STAT6 ChIP-seq and ChIP-qPCR experiments and participated in the interpretation of their biological results. ST participated in the interpretation of the biological results. RL conceived, designed and supervised the STAT6 experiments, introduced the biological problem and participated in the interpretation of the biological results. TA participated in the design and evaluation of the computational study and in drafting the manuscript. LLE conceived, designed and supervised the computational study, participated in the computational tests and their evaluation, and drafted the manuscript. All the authors read and approved the final manuscript.

## Supplementary Material

Additional file 1 **The qPCR-validated regions in STAT6 study**. A table of the regions selected for qPCR validation in the STAT6 study. The presence of the STAT6 binding motif (V$STAT6.01) was determined with the Genomatix MatInspector tool using Matrix Library 8.1 and optimized matrix similarity (default settings). Click here for file
