# Supplementary material for: A practical comparison of methods for detecting transcription factor binding sites in ChIP-seq experiments
Source: BMC Genomics. 2009 Dec 18;10:618. doi: 10.1186/1471-2164-10-618 (PMC2804666; doi:10.1186/1471-2164-10-618)
Supplement: Additional file 1 — The qPCR-validated regions in STAT6 study. A table of the regions selected for qPCR validation in the STAT6 study. The presence of the STAT6 binding motif (V$STAT6.01) was determined with the Genomatix MatInspector tool using Matrix Library 8.1 and optimized matrix similarity (default settings). [file 1471-2164-10-618-S1.PDF]

Laajala TD, Raghav S, Tuomela S, Lahesmaa R, Aittokallio T, Elo LL: **A practical comparison of methods for detecting transcription factor binding sites in ChIP-seq experiments**

Supplementary table 1 - The qPCR-validated regions in STAT6 study

The presence of the STAT6 binding motif (V$STAT6.01) was determined with the Genomatix MatInspector tool using Matrix Library 8.1 and optimized matrix similarity (default settings).

**Chromosome**	**ChIP-seq maximum read overlap in ChIP sample**	**ChIP-seq maximum read overlap in control sample**	**Confirmed with qPCR**	**Genomic location**	**Presence of STAT6 binding motif**
Chr11	16	1	yes	intragenic	yes
Chr8	10	1	yes	<10kb upstream	yes
Chr16	8	1	yes	<10kb upstream	no
Chr3	7	2	yes	<10kb upstream	no
Chr16	7	1	yes	<10kb upstream	yes
Chr6	6	1	yes	intragenic	yes
Chr21	6	1	yes	intragenic	yes
Chr3	6	0	no	intragenic	no
Chr3	5	1	yes	intragenic	no
Chr4	5	1	yes	<10kb upstream	yes
Chr8	5	1	yes	intergenic	yes
Chr19	5	0	yes	<10kb upstream	no
Chr14	5	0	no	intragenic	yes
Chr15	5	1	no	intragenic	no
Chr6	4	0	yes	intragenic	no
Chr8	4	1	yes	intragenic	yes
Chr16	4	1	yes	intragenic	yes
Chr16	4	1	yes	intragenic	yes
Chr10	3	2	yes	intragenic	yes
Chr16	3	0	yes	intragenic	yes
Chr10	3	0	no	intergenic	no
Chr2	2	3	no	<10kb upstream	no
Chr10	0	0	no	intragenic	no
Chr12	0	0	no	intragenic	no
Chr20	0	0	no	intragenic	yes
